# An epilepsy detection method based on multi-dimensional feature extraction and dual-branch hypergraph convolutional network

**DOI:** 10.3389/fphys.2024.1364880

**Published:** 2024-04-12

**Authors:** Jiacen Liu, Yong Yang, Feng Li, Jing Luo

**Affiliations:** ^1^ Chongqing Institute of Green and Intelligent Technology, Chinese Academy of Sciences, Chongqing, China; ^2^ Chengdu Institute of Computer Application, Chinese Academy of Sciences, Chengdu, Sichuan, China; ^3^ Faculty of Mechanical and Electrical Engineering, Kunming University of Science and Technology, Kunming, China; ^4^ Chongqing School, University of Chinese Academy of Sciences, Chongqing, China; ^5^ Department of Neurology, The First Affiliated Hospital of Chongqing Medical University, Chongqing, China

**Keywords:** epileptic seizure detection, EEG, PSD, Conv-LSTM, hypergraph learning

## Abstract

Epilepsy is a disease caused by abnormal neural discharge, which severely harms the health of patients. Its pathogenesis is complex and variable with various forms of seizures, leading to significant differences in epilepsy manifestations among different patients. The changes of brain network are strongly correlated with related pathologies. Therefore, it is crucial to effectively and deeply explore the intrinsic features of epilepsy signals to reveal the rules of epilepsy occurrence and achieve accurate detection. Existing methods have faced the following issues: 1) single approach for feature extraction, resulting in insufficient classification information due to the lack of rich dimensions in captured features; 2) inability to deeply analyze the essential commonality of epilepsy signal after feature extraction, making the model susceptible to data distribution and noise interference. Thus, we proposed a high-precision and robust model for epileptic seizure detection, which, for the first time, applies hypergraph convolution to the field of epilepsy detection. Through a hypergraph network structure constructed based on relationships between channels in electroencephalogram (EEG) signals, the model explores higher-order characteristics of epilepsy EEG data. Specifically, we use the Conv-LSTM module and Power spectral density (PSD), a two-branch parallel method, to extract channel features from space-time and frequency domains to solve the problem of insufficient feature extraction, and can adequately describe the data structure and distribution from multiple perspectives through double-branch parallel feature extraction. In addition, we construct a hypergraph on the captured features to explore the intrinsic features in the high-dimensional space in an attempt to reveal the essential commonality of epileptic signal feature extraction. Finally, using the ensemble learning concept, we accomplished epilepsy detection on the dual-branch hypergraph convolution. The model underwent leave-one-out cross-validation on the TUH dataset, achieving an average accuracy of 96.9%, F1 score of 97.3%, Pre of 98.2% and Re of 96.7%. In addition, the model was generalized performance tested on CHB-MIT scalp EEG dataset with leave-one-out cross-validation, and the average ACC, F1 score, Pre and Re were 94.4%, 95.1%, 95.8%, and 93.9% respectively. Experimental results indicate that the model outperforms related literature, providing valuable reference for the clinical application of epilepsy detection.

## 1 Introduction

The human brain contains approximately 100 billion neurons, making it the most complex organ in the human body. The brain organizes itself through various means such as synaptic connections, forming a complex neural network that dominates our consciousness and behavior (Poo et al., 2016; Sala et al., 2022; Thiebaut de Schotten and Forkel, 2022). Increasing research findings have indicated that the implementation of advanced human functions relies on the connections and communication between different brain regions (Sporns et al., 2005; Yan et al., 2019). In other words, the realization of brain functions primarily depends on highly complex interactions between different areas of the brain in a large-scale network. As a result, the concept of the brain as a network is gaining widespread attention from researchers and clinical practitioners. Currently, the study of interregional relationships in the brain relies heavily on functional connectivity analysis. This method involves analyzing the correlation of neurophysiological activities between brain regions in terms of temporal and frequency domains. It provides objective quantification and interpretable metric information, contributing to the understanding of cognitive function principles and the detection of neurological disorders.

Epilepsy, as a typical neurological disorder, is caused by abnormal neuronal discharges in the brain, leading to a disruption of neural functions. The pathogenesis of epilepsy has been confirmed to be associated with abnormalities in functional connections between relevant brain regions (Shuting et al., 2019; van den Heuvel and Hulshoff Pol, 2010). Neuroscientists are paying attentions to the tools and concepts of network science on a widespread basis, applying them to researches in brain science. These tools and concepts allow for a consistent description and interpretation of interactions among various neural systems within the complex topology of the brain and its networks. This approach has been proven to be successful in systems biology and social network analysis (Michel et al., 2004; Sakkalis, 2011; Fornito et al., 2016; Presigny and De Vico Fallani, 2022).

For the collection of electroencephalographic (EEG) signals, the commonly employed method is electroencephalogram (EEG). EEG is a technique that utilizes electrophysiological metrics to record brain activities. It captures the electrical wave changes during brain activity, serving as a comprehensive reflection of the electrophysiological activity of brain neurons on the cerebral cortex or the surface of the scalp. In recent years, the use of EEG for epileptic seizure detection has drawn widespread attention in the academic circle due to its advantages of easy collection, affordability, and high temporal resolution of data. The installation of EEG collecting channels follows the international 10–20 system, which provides precise positions for channel installation. Each channel’s location corresponds to specific brain regions, facilitating the possibility of analyzing interregional relationships in further analysis (Ein Shoka et al., 2023).

The key innovations of this study include:

(1) for the first time, proposing the neural network model that is based on hypergraph convolution and suitable for epilepsy detection. The model extracted features from each channel by using Conv-LSTM module and PSD, constructed hypergraphs respectively based on the extracted features, and then realized automatic epilepsy detection by adopting hypergraph convolution.
(2) conducting comprehensive experimental tests on the TUH epilepsy dataset and the CHB-MIT scalp EEG dataset to validate the model’s performance. The result indicates that the model can achieve optimal detection performance in epilepsy detection tasks. This approach provides valuable reference for clinical epilepsy detection.

This study is structured as the following: Part 1 provides an overview. Part 2 introduces relevant technologies in epilepsy detection. Part 3 proposes the epilepsy detection model based on multi-dimensional feature extraction and dual-branch hypergraph convolutional network. Part 4 presents the comparative experiments with relevant literature by adopting benchmark dataset. Part 5 discusses the model’s superiority through ablation experiments and various parameter configurations. Part 6 gives the conclusion.

## 2 Related works

Regarding the task of neurological disease detection, existing detection methods are often as the following: 1) Artificial feature extraction is adopted, and the extracted features are calculated by filtering and energy evaluation algorithms such as Multi-variable Fast Iterative Filter (MFIF) (Sharma et al., 2023) and dynamic approximate entropy (Zhang et al., 2023). Then the data is classified by machine learning, for example, decision tree classifier (Nithya et al., 2023) and random forest (Sharma et al., 2018). 2) Applying deep learning model to automatically extract features and performing classification.

In the application of epilepsy detection, some researchers used individual patients’ historical data to train models and then applied these models to test new data from the same patients. Representative achievements in this area include: Hu et al. (2020) proposed an epileptic seizure detection method based on the deep bidirectional long short-term memory (Bi-LSTM) network, achieving an average sensitivity of 93.61% and an average specificity of 91.85% on the long-term scalp EEG database. To address the challenge of limited data samples in individual patient detection tasks, Yang et al. (2022) introduced a specific patient epilepsy detection and analysis method based on data augmentation and deep learning, this approach achieved an average accuracy of 95.47%, an average sensitivity of 93.89%, and an average specificity of 96.48% respectively on the CHB-MIT dataset. Zhou et al. (2018) proposed an epileptic seizure recognition model based on convolutional neural networks (CNN), achieving an average accuracy of 97.5% on the CHB-MIT dataset. Poorani and Balasubramanie (2023) presented a one-dimensional CNN model and a hybrid CNN-LSTM model, where the one-dimensional CNN model achieved an average accuracy of 91.50%, and the CNN-LSTM model achieved an average accuracy of 92.11% on the CHB-MIT dataset. Similarly, Wang et al. (2023) also used the persistent homology method to calculate the complex filter bar code of virtual reality on the CHB-MIT dataset to extract topological features and input them into GoogLeNet for classification. The average accuracy, sensitivity and specificity were 97.05%, 96.71%, and 97.38%, respectively. However, these methods are limited to training and testing on individual subjects, having poor model generalization.

Another research approach involves designing network models with generalization capabilities and utilizing leave-one-out cross-validation. Specifically, this method involves partitioning epilepsy datasets with data from multiple patients. One patient’s data is selected for testing, while the data from other patients are used for training. This approach enhances the model’s generalization capability. Representative achievements include: Zhang et al. (2020) employed feature separation and adversarial representation learning to decompose data into category (seizure and normal) relevant features and patient-specific features, achieved an average accuracy of 80.5% on the TUH EEG dataset. Dissanayake et al. (2021) utilized CNN network structure and Siamese network structure, achieved an accuracy of 88.81% on the CHB-MIT dataset. Yang et al. (2023) applied feature separation adversarial training, achieved an average accuracy of 85.7% on the TUH EEG dataset.

The two aforementioned approaches involve overall feature extraction from a data segment without capturing information transmitting among channels. However, for epilepsy EEG data, the interregional relationships in the brain are highly relevant to seizure patterns, which includes higher-order information of EEG signals and holds important reference significance for epilepsy detection. In the exploration of advanced network feature information from data, researchers have conducted extensive work. Feng et al. (2019a) were among the pioneers who introduced hypergraph neural networks, while Yadati et al. (2019) proposed hypergraph convolutional networks. Jiang et al. (2019) introduced a dynamic hypergraph convolutional neural network, this network utilizes KNN and K-Means to dynamically update the hypergraph structure, enhancing its ability to capture data relationships, it can extract both partial and overall relationships within the data.

In the field of brain science research, some researchers have proposed using graph models to describe pairwise relationships among multi-channel EEG signals. For instance, Zhang et al. (2019) introduced a graph-based hierarchical model that classifies motor intentions based on the relationships between EEG signals and their spatial information. Li et al. (2023) proposed a spatial-temporal hypergraph convolutional network (STHGCN) to capture higher-order relationships in EEG emotion recognition, achieved leading results on the SEED and SEED-IV datasets. Recently, Wagh and Varatharajah (2020) employed graph convolutional neural networks (GCNN) for the classification of epilepsy and normal data, achieving an AUC of 0.90. Currently, there isn’t related research found regarding the application of hypergraph convolution in the field of epilepsy detection. Therefore, taking use of hypergraph convolution can be considered as an important research approach for exploring higher-order information among brain regions in epilepsy patients.

## 3 Methodology

**FIGURE 1:**
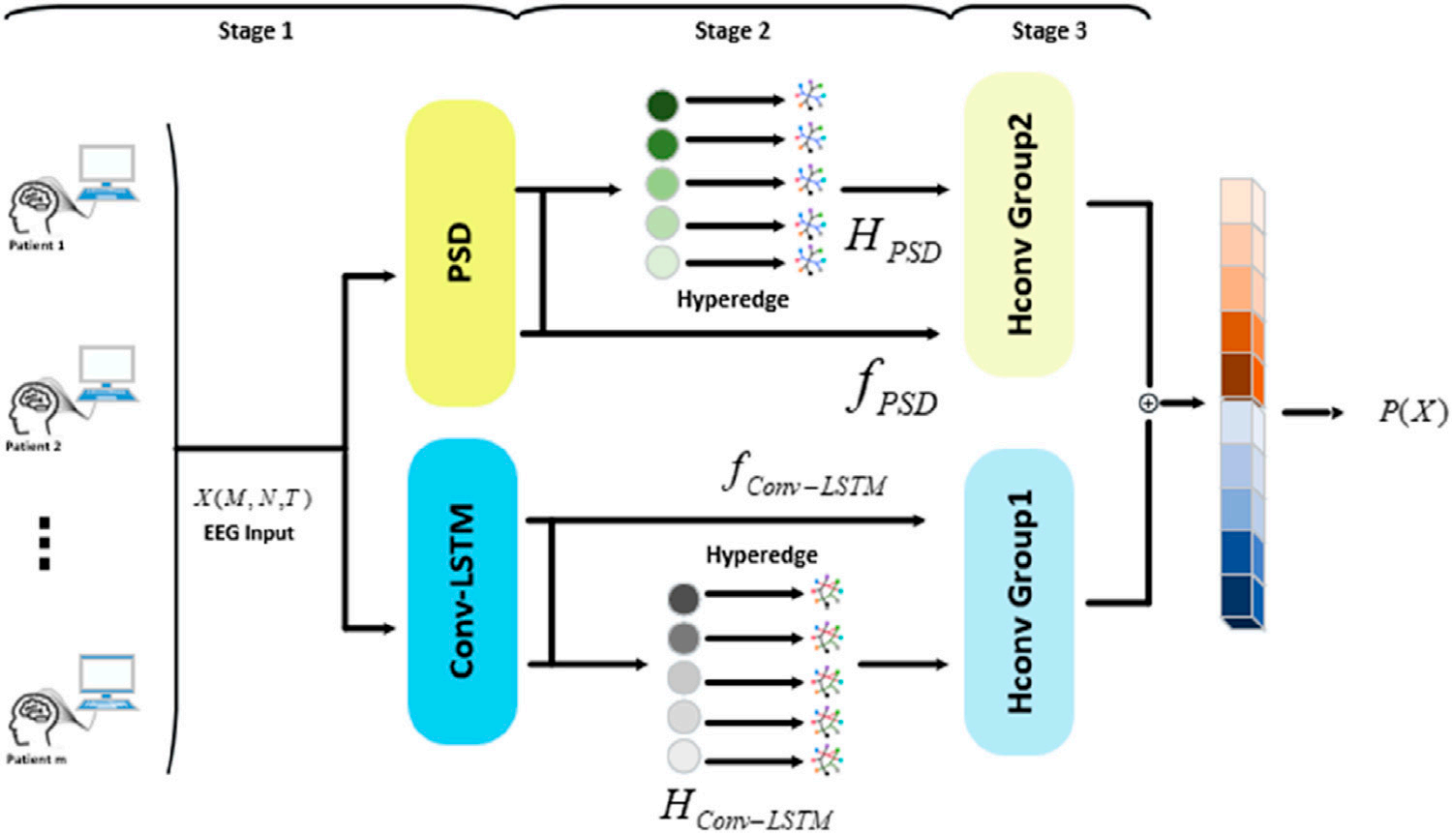
Model structure.

**FIGURE 2:**
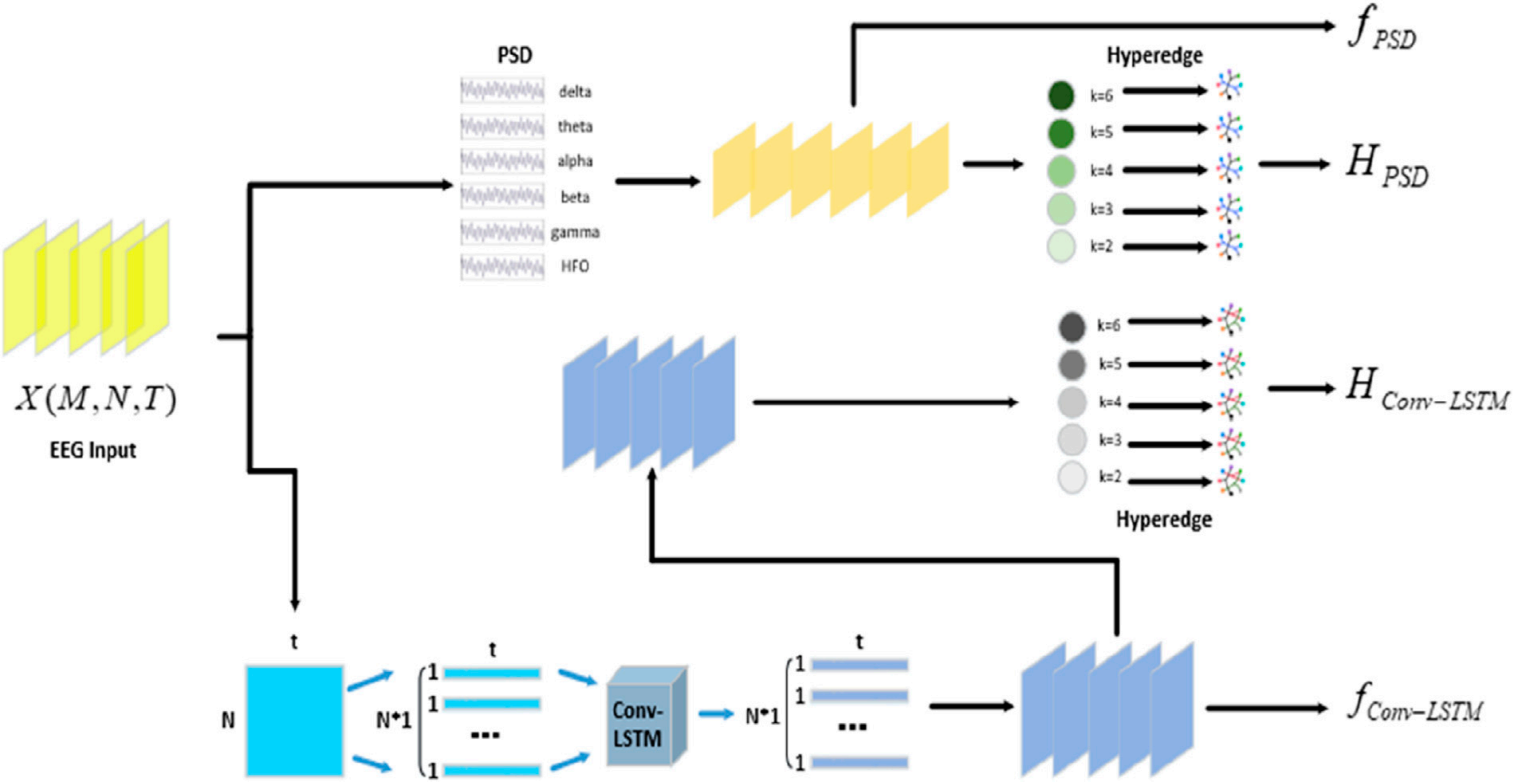
The process of feature extraction stage.

In the study, we proposed an epilepsy detection model based on hypergraph convolution, as illustrated in Figure 1. The processing flow of the model consisted of three stages: 1) feature extraction stage, 2) hypergraph construction stage, and 3) hypergraph convolution stage. The approach in feature extraction stage was depicted in Figure 2. To thoroughly extract multidimensional features from the data, two parallel extraction methods were employed. PSD was used to extract spectral features, and Conv-LSTM neural network was utilized to capture spatiotemporal features. In the hypergraph construction stage, a hypergraph was generated by combining multidimensional features. Hyperedges were adopted to characterize the vertices connected to them, encoding high-order feature information to represent complex data structures in a more flexible manner. In the hypergraph convolution stage, a hypergraph spectral domain convolution method was applied to thoroughly extract high-order data features from epilepsy data, thereby enhancing the model’s generalization capability and classification performance.

### 3.1 Data preprocessing

The EEG is a bioelectrical signal generated by brain activity, which is characterized by uncertainty and randomness. Therefore, prior to analyzing raw data, preprocessing is necessary to eliminate the negative impact of different units and numerical ranges between features on subsequent data analysis. Additionally, it is helpful to improve data quality by adopting various interference elimination techniques, thus to enhance the accuracy of later analyses. This paper adopted min-max regularization technology (Sola and Sevilla, 1997) to regularize the EEG data. The min-max regularization method was shown as the formula (1):

$$X=\frac{X_{i}-X_{\min}}{X_{\max}-X_{\min}}$$ (1)

where, $X_{i}$ represented the original data, X represented the data after data regularization. $X_{\min}$ and $X_{\max}$ represented the minimum and maximum values in the original data.

After regularization of EEG data, in order to extract the features of EEG data, the EEG data were processed in segments. In the study, $X\left(M,N,T\right)$ represented the collected EEG data, where $M=\left\{1,2,\cdots ,m\right\}$ denoted the m−th subject, $N=\left\{1,2,\cdots ,n\right\}$ denoted the n−th signal channel, and T denoted the length of original data. Additionally, for the purpose of facilitating feature extraction, the original data was segmented with a length of t . Then, the original data consisted of S=T/t EEG data segments, with each signal segment s∈S being represented as $x\left(m,s,n,t\right)$ .

### 3.2 Feature extraction

For data feature extraction, we proposed a dual-branch epilepsy feature extraction method in the study. It utilizes PSD to extract spectral features and the Conv-LSTM neural network to capture spatiotemporal features. By extracting data features from multiple dimensions, this approach can provide more information for hypergraph construction, ensuring that the model achieves higher classification accuracy.

#### 3.2.1 Feature extraction using PSD

PSD is a method for calculating the energy distribution of EEG signals at different frequencies from a frequency domain perspective. It helps reveal the essence of brain activity and function. Currently, the frequency domain features of EEG signals represent the most intuitive and convenient characteristics. They are widely utilized in the diagnosis and treatment of neurological disorders. By implementing this algorithm, it is possible to thoroughly extract the energy information of EEG data at different frequency bands, analyzing subjects’ changes in energy across different stages and frequency bands. Consequently, this provides reliable clues for the diagnosis and treatment of relevant neurological disorders.

For the process of extracting PSD features, the first step involved applying Fast Fourier Transform (FFT) to the input data $x\left(m,s,n,t\right)$ (Yudhana et al., 2020), which was defined as Eq. (2):

$$X_{FFT}\left(f\right)=FFT\left(Ham,sft,x\right)$$ (2)

where, FFT represented the Fast Fourier Transform operator, Ham denoted the Hamming window, and sft denoted the frequency domain sampling rate.

Then, performed PSD calculation as Eq. (3) on the obtained $X_{FFT}\left(f\right)$ (Alam et al., 2021):

$$PSD\left(x\right)=\frac{{\left|X_{FFT}\left(f\right)\right|}^{2}}{L}$$ (3)

where, L represented the length of signal. To extract the energy distribution features of data in different frequency bands, we extracted features from the following six sub-frequency bands: δ (0–4 Hz), θ (4–8 Hz), α (8–13 Hz), β (13–30 Hz), γ (30–80 Hz) and high-frequency oscillations HFO (80 Hz–120 Hz), obtaining the power spectral features $P=\left\{p_{\delta },p_{\theta },p_{\alpha },p_{\beta },p_{\gamma },p_{HFO}\right\}$ of the input data $x\left(m,n,t\right)$ .

By integrating, we obtained the PSD features $f_{PSD}\in R^{M\times S\times N\times 6}$ for M subjects, all segments S, channels N, and 6 sub-frequency bands.

#### 3.2.2 Feature extraction using Conv-LSTM

EEG signals are temporal signals, and solely capturing PSD features may not sufficiently acquire the spatiotemporal features of the signal. In the study, we employed the Conv-LSTM spatiotemporal convolutional network (Shi et al., 2015) to automatically extract one-dimensional spatial features and temporal features from EEG signals. This approach allows for a more comprehensive and in-depth extraction of EEG signal features, thereby enhancing the detection accuracy of the model.

In the study, we adopted LSTM networks, which are powerful in representing the extracted temporal domain features, and used the states and outputs of the network’s memory cells at each time step, to construct spatial convolutional norms learning and the sequential accumulation of effective signal features. Therefore, between adjacent time steps, parameters are selectively inherited, aiding in the construction of contextual information and ensuring the integrity of feature structures presentation.

In the current time segment S , the state and output of the LSTM memory cell were represented by $C_{s}$ and $f_{s-1}$ respectively, while $C_{s-1}$ and $f_{s-1}$ represented the state and output of the cell in the previous time segment s−1 . The calculation process for extracting features from a single-channel $x\left(m,s,1,t\right)$ input was defined as Eqs (4)–(7):

$$h_{s}=\sigma \left(\psi_{1}^{3\times 3}\left(f_{s-1}⊕x\left(m,1,t\right)\right)\right)$$ (4)

$${\tilde{C}}_{s}=\sigma \left(\psi_{2}^{3\times 3}\left(f_{s-1}⊕x\left(m,1,t\right)\right)\right)$$ (5)

$$C_{s}=h_{s}\circ \left({\tilde{C}}_{s}+C_{s-1}\right)$$ (6)

$$f_{s}=h_{s}\circ C_{s}$$ (7)

where, ∘ and ⊕ represented Hadamard product and dimension concatenation respectively, $\psi_{1}^{3\times 3}$ and $\psi_{2}^{3\times 3}$ represented the feature tensor convolution and state tensor convolution for 3×3 respectively. The output of the spatiotemporal convolution branch $f_{Conv-LSTM}\in R^{M\times S\times N\times t}$ was obtained by extracting features from all channels.

### 3.3 Hypergraph construction

Building on the foundation of feature extraction, we constructed a hypergraph $G^{F}=\left(V,E,W\right)$ based on the distance relationships between features, with $F\in \left\{f_{Conv-LSTM},f_{PSD}\right\}$ being the extracted feature set. Each EEG data channel serving as a vertex ν∈V in the hypergraph and each hyperedge e∈E being formed by connecting a vertex ν with its k -nearest neighboring vertices at the minimum Euclidean distance. Among which $k\in \left\{2,3,4,5,6\right\}$ , five hypergraph adjacency matrices were formed. Each hyperedge e∈E contained two or more vertices and was assigned a positive weight $W_{e}$ , forming a diagonal matrix $W^{N\times N}$ from integrating all the weights. A hypergraph $G^{F}=\left(V,E,W\right)$ can be equivalently represented by $\left|V\right|\times \left|E\right|$ to be an adjacency matrix $H\in R^{\left|V\right|\times \left|E\right|}$ , with entries defined as Eq. (8) (Feng et al., 2019b):

$$h\left(\nu ,e\right)=\left\{\begin{array}{l} 1,\nu \in e\\ 0,\nu \notin e \end{array}\right.$$ (8)

For any given feature vertex ν∈V , its degree was expressed as Eq. (9):

$$d\left(\nu \right)=\sum_{e\in E}w\left(e\right)h\left(\nu ,e\right)$$ (9)

where, $w\left(e\right)$ represented the weight corresponding to the hyperedge e .

For any given hyperedge e∈E , the expression $d\left(e\right)$ for its degree was defined as Eq. (10):

$$d\left(e\right)=\sum_{\nu \in V}h\left(\nu ,e\right)$$ (10)

All expressions for the degrees of feature vertices ν and hyperedges e were recorded in diagonal matrices $D_{v}\in R^{\left|V\right|\times \left|V\right|}$ and $D_{e}\in R^{\left|E\right|\times \left|E\right|}$ . $H_{PSD}$ , $D_{\nu }^{PSD}$ and $D_{e}^{PSD}$ obtained by constructing hypergraphs through PSD features; while $H_{Conv-LSTM}$ , $D_{\nu }^{Conv-LSTM}$ and $D_{e}^{Conv-LSTM}$ obtained by constructing hypergraphs through spatiotemporal convolution features.

### 3.4 Hypergraph convolution

Based on the H , $D_{v}$ and $D_{e}$ generated during the hypergraph construction process, as well as the subject features input $f_{Conv-LSTM}\in R^{M\times S\times Num1}$ and $f_{PSD}\in R^{M\times S\times Num2}$ , where Num1=N×t represented the feature dimension of the spatiotemporal convolution branch and Num2=N×6 represented the feature dimension of the PSD branch, hypergraph convolutions were conducted for each branch (Li et al., 2023), which were defined as Eq. (11). The construction process is shown in Algorithm 1.

Algorithm 1 **The Proposed Hypergraph Convolution Approach.** 
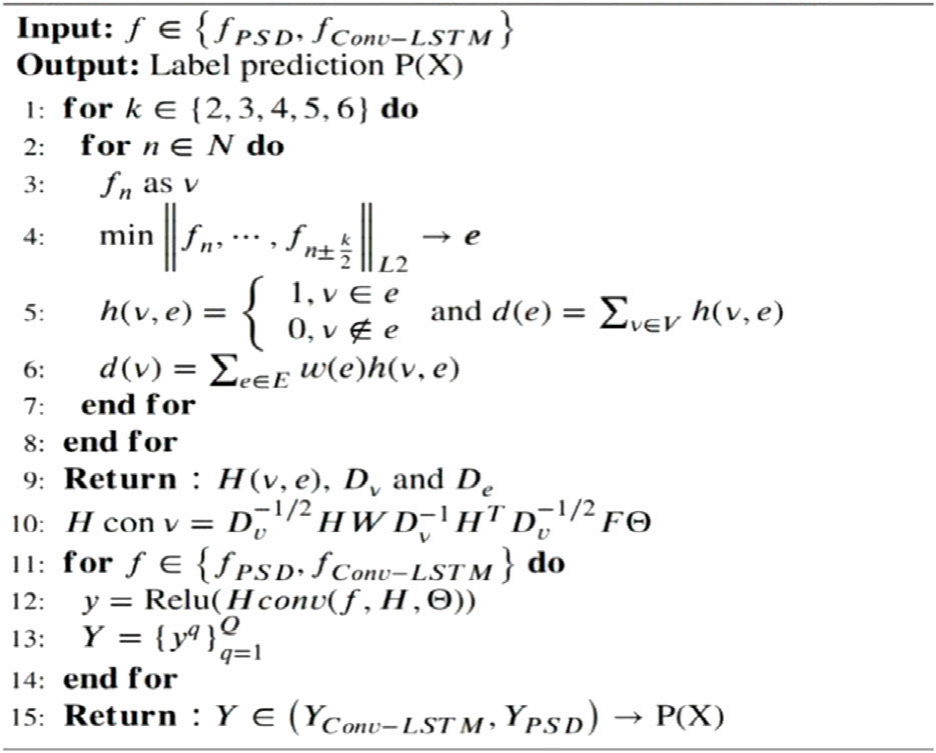

$$Hconv=D_{\nu }^{-1/2}HWD_{e}^{-1}H^{T}D_{\nu }^{-1/2}F\Theta$$ (11)

where, $\Theta \in R^{Num\times Num}$ represented the model parameters updated during the training process through backpropagation of the cross-entropy loss on training data. For the spatiotemporal convolution branch after Relu mapping, the result of the hypergraph convolution was defined as Eq. (12):

$$y_{Conv-LSTM}=\text{Re}lu\left(Hconv\left(f_{Conv-LSTM},H_{Conv-LSTM},\Theta \right)\right)$$ (12)

For the PSD branch, the hypergraph convolution after Relu mapping was defined as Eq. (13):

$$y_{PSD}=\text{Re}lu\left(Hconv\left(f_{PSD},H_{PSD},\Theta \right)\right)$$ (13)

To enhance the model’s generalization and robustness, we have introduced a convolutional expansion factor Q, expanding the feature output to $Y_{Conv-LSTM}={\left\{y_{Conv-LSTM}^{q}\right\}}_{q=1}^{Q}$ and $Y_{PSD}={\left\{y_{PSD}^{q}\right\}}_{q=1}^{Q}$ .

Through the dual-branch hypergraph convolution, we obtained the frequency domain features and spatiotemporal features representations of the data respectively. These two representations were then connected in a cascaded manner. Finally, label prediction was achieved through two convolutional layers $\psi^{1\times 1}$ for 1×1 and the softmax function was defined as Eq. (14):

$$P\left(X\right)=soft\max\left(\psi^{1\times 1}\left(\psi^{1\times 1}\left(Y_{Conv-LSTM}⊕Y_{PSD}\right)\right)\right)$$ (14)

To complete the model training, we introduced the cross-entropy loss function in the study. The cross-entropy loss function measured the difference between the predicted probability distribution of the proposed model and the true probability distribution. During backpropagation, gradients were used to constrain the hyperparameters and convolutional parameters in the model, aiming to improve the model’s predictive accuracy. It was specifically represented by Eq. (15):

$$L=-P*\log\left(y_{label}\right)-\left(1-P\right)*\log\left(1-y_{label}\right)$$ (15)

where, $P\in \left(0,1\right)$ represented the predicted probability of the network, and $y_{label}$ denoted the data label.

## 4 Experiments

### 4.1 Datasets and evaluation metrics

The proposed model in the study was extensively evaluated on the publicly available dataset, TUH epilepsy dataset (Obeid and Picone, 2016), to thoroughly assess and validate the effectiveness of the model and its components. The dataset included 2,993 records of at least 15 min duration obtained from 2,329 unique patients and consisted of a developed and separate final assessment set. It contains records of male and female patients from a variety of age ranges (7 days −96 years), and therefore includes infants, children, adolescents, adults, and elderly patients. Pathologies diagnosed in patients in the dataset include (but are not limited to) epilepsy, stroke, depression, and Alzheimer’s disease, however, only binary labels are provided. The dataset includes physician reports that provide additional information about each EEG record, such as major EEG findings, the patient’s ongoing medication, and medical history. In the description of the dataset, TUH reported an inter-rater confidence of 97%–100%. In the literature, the reported scores are usually much lower. The nearly perfect rating may be the result of a review of the survey results by medical students who were aware of the diagnosis in advance. The dataset followed the international 10–20 system to perform channel installation and data collection, with 21 channels and a sampling rate of 250 Hz. We randomly selected subjects with seizure duration being more than 250 s, forming 14 TUH subsets as the experimental datasets. For each subject, we used 500 s of EEG signals (half normal data and half seizure data), with each EEG segment having 250 sampling points (lasting for 1 s), i.e., t = 250, and adjacent segments overlapping by 50%. For each EEG segment, the seizure state ones being categorized as positive were assigned a label of 1, while the normal state ones being categorized as negative were assigned a label of 0. Then, the 14 TUH subsets were divided into training set and testing set according to leave-one-out cross-validation.

The leave-one-out cross-validation method used in this paper is a special cross-validation method. Specifically, the TUH dataset contains 14 patient data subsets, and through 14 experimental trainings, only 1 patient sample is retained as the validation set each time, and the remaining 13 patients are used as the training set. Because each sample is independently verified, the model is not affected by the division of training set and verification set, and the validity and robustness of experimental data are guaranteed. To be more specific, the designed diagnosis model with robustness must handle both intra-patient factors and inter-patient noise to embrace clinical and more complex situations needs such as patient-independent: the testing patient is unseen in the training stage (Zhang et al., 2020). Therefore, the leave-one-out cross-validation method provides a patient-independent validation of the differences in data structures among patients during training and testing. The high average accuracy obtained by leave-one-out cross-validation method can reflect the anti-noise interference ability and robustness of the proposed model. At the same time, we evaluated the training time and test delay of our method. The results showed that the test required only 0.03 s (0.01 s for EEG decomposition and 0.02 s for attentional epilepsy diagnosis), while a single training session required 1,852.7 s. In conclusion, in a potential online seizure diagnosis system, the diagnostic delay of our approach is acceptable.

At the same time, we used the epilepsy scalp EEG dataset from Boston Children’s Hospital, United States (Goldberger et al., 2000), which is named as CHB-MIT scalp EEG, to further verify the validity of the proposed model. This dataset contains 24 consecutive scalp EEG recordings from 23 patients. The first and 21st records were from the same patient, and the 24th record did not provide personal information. The dataset followed the international 10–20 system to perform channel installation and data collection with a signal sampling frequency of 256 Hz and a resolution of 16 bits per second. In the dataset, 23 channels were used for most records. For the convenience of research, only EEG data containing 23 channels were retained in this paper, and records with channel number less than or greater than 23 would be discarded. The number of channels recorded in the 12th and 15th sections was not sufficient for the requirements of this experiment, so the data recorded in the 12th and 15th sections were discarded. Table 1 shows the personal information and the number of seizures recorded in the CHB-MIT scalp EEG dataset.

$$ACC=\frac{TP+TN}{TP+FP+TN+FN}$$ (16)

$$\mathrm{Pr}e=\frac{TP}{TP+FP}$$ (17)

$$\text{Re}=\frac{TP}{TP+FN}$$ (18)

$$F1=\frac{2\mathrm{Pr}e\cdot \text{Re}}{\mathrm{Pr}e+\text{Re}}$$ (19)

**TABLE 1:** CHB-MIT scalp EEG dataset information.

ID	Gender	Age/year	Number of seizures
1	Female	11	7
2	Male	11	3
3	Female	14	7
4	Male	22	4
5	Female	7	5
6	Female	1.5	10
7	Female	14.5	3
8	Male	3.5	5
9	Female	10	4
10	Male	3	7
11	Female	12	3
13	Female	3	10
14	Female	9	8
16	Female	7	8
17	Female	12	3
18	Female	18	6
19	Female	19	3
20	Female	6	8
21	Female	13	4
22	Female	9	3
23	Female	6	7
24	—	—	16

The performance of the algorithm was evaluated using accuracy (ACC), Precision (Pre), Recall (Re), and F1 in the experiment.

In the above equations from (16) to (19), TP (True Positive) denoted the samples judged as positive that are actually positive, TN (True Negative) denoted the samples judged as negative that are actually negative, FP (False Positive) denoted the samples judged as positive that are actually negative, FN (False Negative) denoted the samples judged as negative that are actually positive.

### 4.2 Benchmark

**FIGURE 3:**
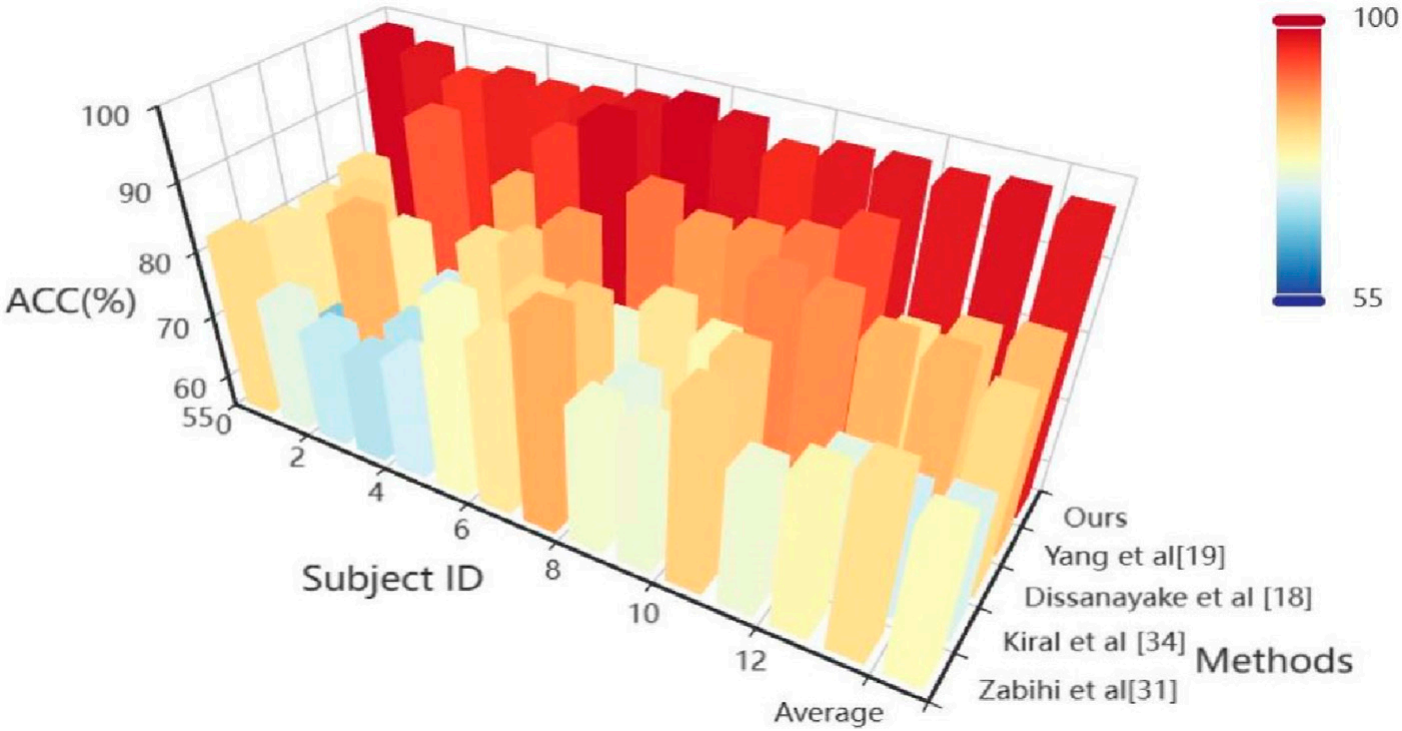
Heatmap visualization results from the comparative experiment.

On the TUH dataset, using the same data segment length and employing leave-one-out cross-validation, we compared our approach with seven other methods. The comparative results of ACC are presented in Table 2 and Heatmap in Figure 3. Meanwhile, the comparison results of multiple indicators are shown in Table 3.

**TABLE 2:** Comparative results.

Methods	Subject ID														Average
	0	1	2	3	4	5	6	7	8	9	10	11	12	13	
Zabihi et al. (2013)	82.1	74.6	71.9	70.6	72.6	77.3	80.4	86.3	76.2	75.8	83.2	75.8	78.4	81.6	77.6
Fergus et al. (2015)	80.3	77.5	86.5	75.1	80.1	71.8	85.3	89.8	72.2	75.8	86.6	72.5	79.1	82.3	79.6
Schirrmeister et al. (2017)	79.3	74.3	96.5	75.8	78.9	66.5	81.3	87.1	61.9	63.4	91.9	57.1	74.4	71.1	76.0
Kiral-Kornek et al. (2018)	80.5	66.9	85.5	70.9	77.2	61.9	82.3	83.6	74.6	59.8	83.5	55.6	74.5	72.6	73.6
Zhang et al. (2020)	84.1	82.6	97.8	77.4	84.2	73.3	91.1	91.4	69.7	65.2	92.3	60.4	77.2	78.7	80.5
Dissanayake et al. (2021)	80.4	83.1	79.2	72.6	81.4	83.4	87.9	75.8	80.8	78.2	89.2	88.5	85.6	85.5	82.3
Yang et al. (2023)	82.0	72.8	92.4	60.4	86.0	94.4	98.4	90.4	87.6	88.4	90.0	93.2	81.2	83.6	85.7
Ours	98.5	97.2	94.8	96.2	95.9	96.5	97.4	98.7	97.7	95.5	96.9	97.2	96.9	97.5	96.9

**TABLE 3:** Comparison results of multiple indexes.

Methods	Evaluation metrics			
	ACC	F1	Pre	Re
Zabihi et al. (2013)	77.6	82.8	81.5	84.1
Fergus et al. (2015)	79.6	83.3	85.4	81.4
Schirrmeister et al. (2017)	76.0	79.4	78.5	81.1
Kiral-Kornek et al. (2018)	73.6	77.0	74.8	78.9
Zhang et al. (2020)	80.5	84.4	86.4	82.6
Dissanayake et al. (2021)	82.3	86.8	89.5	84.9
Yang et al. (2023)	85.7	89.6	90.9	88.1
Ours	96.9	97.3	98.2	96.7

Zabihi et al. (2013) used discrete wavelet transform (DWT) to calculate indicators such as relative scale energy and Shannon entropy as features. Support vector machines are used for data classification.

Fergus et al. (2015) used PSD and calculated metrics such as peak frequency and maximum frequency as features; KNN is used for data classification.

Schirrmeister et al. (2017) used convolutional neural networks to decode task-related information from EEG signals to distinguish epileptic fragments.

Kiral-Kornek et al. (2018) designed deep neural networks for epilepsy diagnosis and further developed predictive systems for wearable devices.

Zhang et al. (2020) propose an adversarial representation learning strategy to achieve robust and interpretable seizure detection.

Dissanayake et al. (2021) used CNN network structure and Siamese network structure to improve the generalization ability of the model.

Yang et al. (2023) used multistage time-spectrum feature extraction network, feature separation network and invariant feature extraction network to extract the essence of features in depth to avoid differences in data distribution between patients.

Through comparative analysis, Schirrmeister (Schirrmeister et al., 2017) and Kiral (Kiral-Kornek et al., 2018) initially applied deep neural networks to epilepsy detection but failed to consider the diversity in patient data. This lack of extracting consistent features from the data negatively impacted the model training, reducing the detection accuracy for new patients. In this context, Zabihi (Zabihi et al., 2013) used relative scale energy and Shannon entropy, etc. as features, and Fergus (Fergus et al., 2015) used peak frequency and maximum frequency, etc. as features, to capture common features in the data. However, these approaches were unable to identify common features at higher-dimensional levels. Therefore, the proposed method in the study demonstrated better detection accuracy comparing to Schirrmeister’s (Schirrmeister et al., 2017) and Kiral’s (Kiral-Kornek et al., 2018). Additionally, shallow feature commonality extraction cannot thoroughly explore the essence of features, resulting in test results lower than those achieved by Zhang (Zhang et al., 2020), Dissanayake (Dissanayake et al., 2021), and Yang (Yang et al., 2023). Zhang (Zhang et al., 2020) and Dissanayake (Dissanayake et al., 2021) employed deep learning methods such as adversarial training and contrastive training, etc., reducing the negative impact of differences in data distribution between patients. Their results were superior to those not considering removing the negative impact of data distribution shift between patients. Furthermore, Yang (Yang et al., 2023) first used a multi-level time-spectrum feature extraction network to capture common features and then input it into a feature separation network and an invariant feature extraction network, achieving more excellent accuracy performance by deeply extracting the essence of features and avoiding differences in data distribution between patients. We introduced in the study, for the first time, a hypergraph convolutional neural network model suitable for epilepsy detection. It captures multidimensional features through parallel dual branches while constructing hypergraph convolution. This exploration of high-order common information between brain regions of epilepsy patients can project essential features of data structure from a higher dimension, thereby reducing the impact of skewed distribution.

Through comprehensive analysis of several evaluation indicators, the proposed model reached 96.9% (ACC), 97.3% (F1), 98.2% (Pre) and 96.7% (Re) on the TUH dataset. Compared with the epilepsy detection study in the preface, Yang’s method has an 11.2% improvement in ACC, 7.7% improvement in F1, 7.1% improvement in Pre and 8.6% improvement in Re (Yang et al., 2023). Compared with the traditional support vector machine method (Zabihi et al., 2013), it has a greater improvement: 19.3%, 14.5%, 16.7%, and 12.6% corresponding to ACC, F1, Pre and Re, respectively. The excellent performance of the proposed model is further described through multiple evaluation dimensions.

On the CHB-MIT scalp EEG dataset, we used the model and the leave-one-out cross-validation method to conduct a full experiment on this dataset. The experimental results (ACC) are shown in Table 4.

**TABLE 4:** Experimental results.

Subject ID							
1	2		3	4	5		6
92.8	90.1		98.3	92.7	91.5		96.3
7	8		9	10	11		13
95	96.4		93.3	98.1	94.5		95.9
14	16		17	18	19		20
91.8	98.2		98.8	96.7	97.5		97.1
21	22		23	24			
92.6	94.8		93.1	97.8			
Evaluation metrics							
Average ACC		Average F1		Average Pre		Average Re	
94.4		95.1		95.8		93.9	

The generalization ability of the proposed model was tested on the CHB-MIT scalp EEG dataset. From the experimental results in the table, it can be concluded that the ACC, F1, Pre and Re of 22 patients evaluated by the model were as high as 94.4%, 95.1%, 95.8%, and 93.9%. Each evaluation index is above 90%, and the comprehensive ability is outstanding. Among them, the EEG test results of patient 17 were as high as 98.8%, and the model’s worst performance was patient 16, at 89.4%. The experimental data of the two datasets show that the proposed model has good generalization ability and robustness.

## 5 Discussions

**FIGURE 4:**
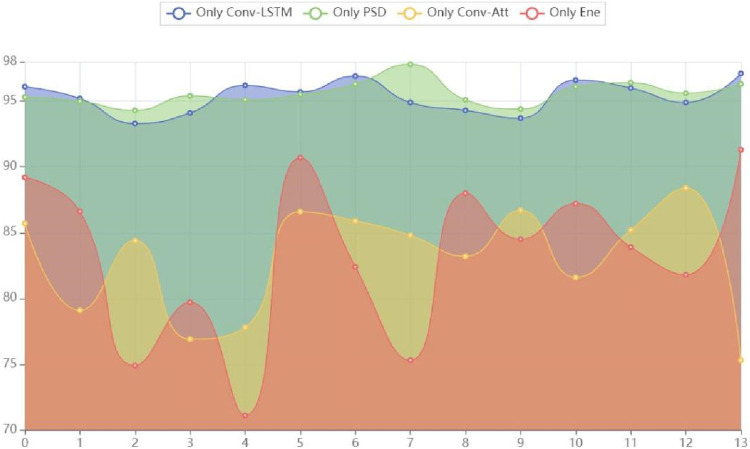
Area of experimental results for branch ablation.

In order to analyze the effectiveness of the proposed method, we conducted extensive ablation experiments on TUH dataset with its components and parameters. First, in the feature extraction stage, to validate the effectiveness of the dual-branch structure, we conducted two sets of experiments: “Only Conv-LSTM,” “Only PSD,” “Only Conv-Att” and “Only Ene,” which respectively represented only using Conv-LSTM to extract features, only using PSD to extract features, using only channel attention convolution and only energy representations. Secondly, for the important parameters $k\in \left\{2,3,4,5,6\right\}$ required for hypergraph construction, we conducted sequential k-value experiments to explore the impact of the hypergraph constructed by different k values, the individual k-value and the combined six k values on the network detection accuracy. All experiments were conducted using leave-one-out cross-validation on the TUH dataset. The ACC results of the branch ablation experiment are shown in Table 5; Figure 4, and the hypergraph parameter experiment results are shown in Table 6.

**TABLE 5:** Experimental results of branch ablation.

Methods	Subject ID														Average
	0	1	2	3	4	5	6	7	8	9	10	11	12	13	
Only Conv-LSTM	96.1	95.2	93.3	94.1	96.2	95.7	96.9	94.9	94.3	93.7	96.6	96	94.9	97.1	95.4
Only PSD	95.3	95	94.3	95.4	95.1	95.5	96.3	97.8	95.1	94.4	96.1	96.4	95.6	96.3	95.6
Only Conv-Att	85.7	79.1	84.4	76.9	77.8	86.6	85.9	84.8	83.2	86.7	81.6	85.2	88.4	75.3	82.9
Only Ene	89.2	86.6	74.9	79.7	71.1	90.7	82.4	75.3	88	84.5	87.2	83.9	81.8	91.3	83.3
All (Conv-LSTM + PSD)	98.5	97.2	94.8	96.2	95.9	96.5	97.4	98.7	97.7	95.5	96.9	97.2	96.9	97.5	96.9

**TABLE 6:** Experimental results of hypergraph parameters.

Methods	Subject ID														Average
	0	1	2	3	4	5	6	7	8	9	10	11	12	13	
k = 2	94.5	91.6	88	89.3	92.1	91.2	90.7	91.6	88.3	92.5	94.3	92.3	91.8	89.5	91.3
k = 3	90.7	87.6	90.1	88.6	89.9	90.6	89.7	91.7	91.1	90.1	93.6	93.8	92.3	90.3	90.7
k = 4	93.6	89.4	91.6	94.5	91.5	93.9	94.7	91.4	90.5	90.9	90.1	94.2	89.1	91.2	91.9
k = 5	94.8	91.1	89.5	90.2	93.2	91.0	92.1	92.5	93.5	91.2	93.4	93.5	91.5	94.4	92.3
k = 6	92.8	94.5	92.8	93.0	92.8	92.6	88.6	92.7	93.8	93.3	93.6	94.7	92.1	93.9	92.9
All	98.5	97.2	94.8	96.2	95.9	96.5	97.4	98.7	97.7	95.5	96.9	97.2	96.9	97.5	96.9

To validate the effectiveness of the proposed dual-branch structure, a comparison between the results of single-branch experiments and dual-branch experiments revealed that the model testing average accuracy was 95.4% when only using Conv-LSTM to extract features, while the model testing average accuracy was 95.6% when only using PSD to extract features. At the same time, “Only Conv-Att” and “Only Ene” performed significantly lower in ACC at 82.9% and 83.3%, respectively, than “Only Conv-LSTM” and “Only PSD.” This shows that the essential ability of hypergraph convolutional representation data constructed after spatial attention extraction branch and energy branch extraction is inferior to Conv-LSTM branch and PSD branch. Therefore, Conv-LSTM branch and PSD branch are selected as more effective feature extraction methods in this paper.

By comparing the model performance of using only the Conv-LSTM feature extraction branch and using only the PSD feature extraction branch, it was found that the model performance using only the PSD feature extraction branch was superior to the model performance using only the Conv-LSTM feature extraction branch. We conducted a model performance comparison using an area chart, as shown in Figure 2, where the model performance using only the PSD feature extraction branch dominated in total area, highlighting its outstanding testing performance. The reason for this result may be that frequency domain features have advantages in explaining the essence of epilepsy EEG information, while spatiotemporal domain information is more focused on connecting temporal information to construct context. Therefore, after extracting frequency domain features, the fusion of temporal information can effectively reveal multiple aspects of the essential characteristics of epilepsy EEG signals, achieving a superior detection accuracy of 96.9%.

Upon completing the dual-branch feature extraction, it is crucial to effectively construct the hypergraph for feature representation and hypergraph learning. In hypergraph construction, each EEG data channel served as a vertex ν∈V in the hypergraph, and each hyperedge e∈E was formed by connecting a vertex ν with its k -nearest neighboring vertices at the minimum Euclidean distance. The parameter k determined the number of adjacency matrices and the dimensionality of hypergraph information, reflecting the quality of hypergraph construction and the richness of classification information. To investigate the impact of different $k\in \left\{2,3,4,5,6\right\}$ on model performance, experiments were conducted. By comparing experiment results with different k value: ACC (k = 2) = 91.3%, ACC (k = 3) = 90.7%, ACC (k = 4) = 91.9%, ACC (k = 5) = 92.3%, ACC (k = 6) = 92.9%, ACC [k= (Sporns et al., 2005; Shuting et al., 2019; Yan et al., 2019; Sala et al., 2022; Thiebaut de Schotten and Forkel, 2022)] = 96.9%, it was observed that ACC (k = 6) = 92.9% outperformed ACC (k = 2) = 91.3%. During the hypergraph construction process, this occurred due to that the more adjacent vertices ν∈V found by the minimum Euclidean distance allows the hypergraph to contain more feature information, and the constructed hyperedges can better reflect common features. However, the situation as ACC (k = 3) = 90.7% being lower than ACC (k = 2) = 91.3% also existed. This could be because: for the three adjacency matrices constructed via the three nearest neighboring vertices (searched through the KNN algorithm), comparing with k = 2, the additionally constructed adjacency matrix may introduce distant noise information for the original vertex, which did not effectively contribute to the construction of the hypergraph for the original vertex. To effectively mitigate the impact of the k value on the model’s performance, we employed multiple k values in the study to construct hypergraphs respectively and adopted a systematic ensemble approach, resulting in ACC [k= (Sporns et al., 2005; Shuting et al., 2019; Yan et al., 2019; Sala et al., 2022; Thiebaut de Schotten and Forkel, 2022)] = 96.9%.

In future study, we propose three recommendations as the following:

Firstly, the proposed method employed a dual-branch parallel extraction and hypergraph learning structure, capturing frequency domain information and spatiotemporal domain information respectively. Next, we can increase the data volume in parallel branches to extract more discriminative features from multiple dimensions, thereby enhancing the model’s performance.

Secondly, in the hypergraph construction stage, the current approach only utilized the KNN method to search for vertices and construct hyperedges. Next, we can explore various ways of constructing hypergraphs and integrate them to enhance the feature representation capability of hypergraphs.

Lastly, the proposed method was trained and tested only on two public datasets, lacking validation on real clinical datasets. Therefore, we will conduct validation on the actual performance of the model using clinical data in the future.

## 6 Conclusion

In the study, we have first ever introduced a novel neural network model for epilepsy detection based on hypergraph convolution. Addressing the insufficient feature extraction in traditional methods for epilepsy datasets, which fails to deeply reveal the high-order characteristics of seizure data, we have proposed the dual-branch approach to extract features from each channel using Conv-LSTM module and PSD. This has been a highly effective way to explore both the frequency domain features and spatiotemporal domain features information of epilepsy signals. Based on this, hypergraphs were constructed using the KNN algorithm, exploring the commonalities and intrinsic information of epilepsy data in the hypergraph structure. Finally, hypergraph convolution was applied to achieve graph feature extraction and automatic epilepsy detection. In the testing and validation phase, we conducted leave-one-out cross-validation with 14 patients’ data selected from the TUH dataset according to experimental requirements and compared the results with relevant literature. The proposed method achieved the best results. In addition, the effectiveness and generalization ability of the proposed model are verified on CHB-MIT Scalp EEG dataset. It indicates that the high-order hypergraph features, which the model explores, are highly discriminative, being able to achieve higher detection accuracy and provide valuable reference for the clinical application of epilepsy detection.

## Data Availability

Publicly available datasets were analyzed in this study. This data can be found here: I. Obeid and J. Picone, “The Temple University Hospital EEG Data Corpus,” *Frontiers in Neuroscience*, vol. 10, p. 196, 2016.

## References

[B1] Alam M. N. Ibrahimy M. I. Motakabber S. M. A. (2021). in Feature extraction of EEG signal by power spectral density for motor imagery based BCI[C]//2021 8th International Conference on Computer and Communication Engineering (ICCCE) (IEEE), 234–237.

[B2] Dissanayake T. Fernando T. Denman S. Sridharan S. Fookes C. (2021). Deep learning for patient-independent epileptic seizure prediction using scalp EEG signals. IEEE Sensors J. 21 (7), 9377–9388. 10.1109/jsen.2021.3057076 34314363

[B3] Ein Shoka A. A. Dessouky M. M. El-Sayed A. Hemdan E. E. D. (2023). EEG seizure detection: concepts, techniques, challenges, and future trends. Multimedia Tools Appl. 82, 42021–42051. 10.1007/s11042-023-15052-2 PMC10071471 37362745

[B4] Feng Y. You H. Zhang Z. (2019a). “Hypergraph neural networks. Biomedical engineering,” in Paper presented at the Proceedings of the AAAI conference on artificial intelligence, 162–175.

[B5] Feng Y. You H. Zhang Z. Ji R. Gao Y. (2019b). “Hypergraph,” in Neural Networks//Proceedings of the AAAI conference on artificial intelligence, 3558–3565. 01. 10.1609/aaai.v33i01.33013558

[B6] Fergus P. Hignett D. Hussain A. Al-Jumeily D. Abdel-Aziz K. (2015). Automatic epileptic seizure detection using scalp EEG and advanced artificial intelligence techniques. Biomed. Res. Int. 2015, 986736. 10.1155/2015/986736 25710040 PMC4325968

[B7] Fornito A. Zalesky A. Bullmore E. (2016). Fundamentals of brain network analysis [M].

[B8] Goldberger A. L. Amaral L. A. N. Glass L. Hausdorff J. M. Ivanov P. C. Mark R. G. (2000). PhysioBank, PhysioToolkit, and PhysioNet: components of a new research resource for complex physiologic signals. circulation 101 (23), e215–e220. 10.1161/01.cir.101.23.e215 10851218

[B9] Hu X. Yuan S. Xu F. Leng Y. Yuan K. Qi Y. (2020). Scalp EEG classification using deep Bi-LSTM network for seizure detection. Comput. Biol. Med. 124, 103919. 10.1016/j.compbiomed.2020.103919 32771673

[B10] Jiang J. Wei Y. Feng Y. Cao J. Gao Y. (2019). “Dynamic hypergraph neural networks[C]//IJCAI,” in Proceedings of the Twenty-Eighth International Joint Conference on Artificial Intelligence Maintrack, 2635–2641. 10.24963/ijcai.2019/366

[B11] Kiral-Kornek I. Roy S. Nurse E. Mashford B. Karoly P. Carroll T. (2018). Epileptic seizure prediction using big data and deep learning: toward a mobile system. EBioMedicine 27, 103–111. 10.1016/j.ebiom.2017.11.032 29262989 PMC5828366

[B12] Li M. Qiu M. Zhu L. Kong W. (2023). Feature hypergraph representation learning on spatial-temporal correlations for EEG emotion recognition. Cogn. Neurodynamics 17 (5), 1271–1281. 10.1007/s11571-022-09890-3 PMC10542078 37786664

[B13] Michel C. M. Murray M. M. Lantz G. Gonzalez S. Spinelli L. Grave de Peralta R. (2004). EEG source imaging. Clin. Neurophysiol. 115 (10), 2195–2222. 10.1016/j.clinph.2004.06.001 15351361

[B14] Nithya K. Sharma S. Sharma R. R. (2023). in Eigenvalues of Hankel Matrix based Epilepsy Detection using EEG Signals[C]//2023 2nd International Conference on Paradigm Shifts in Communications Embedded Systems, Machine Learning and Signal Processing (PCEMS) (IEEE), 1–6.

[B15] Obeid I. Picone J. (2016). The temple university hospital EEG data corpus. Front. Neurosci. 10, 196. 10.3389/fnins.2016.00196 27242402 PMC4865520

[B16] Poo M. M. Du J. L. Ip N. Y. Xiong Z. Q. Xu B. Tan T. (2016). China brain project: basic neuroscience, brain diseases, and brain-inspired computing. Neuron 92 (3), 591–596. 10.1016/j.neuron.2016.10.050 27809999

[B17] Poorani S. Balasubramanie P. (2023). Deep learning based epileptic seizure detection with EEG data. Int. J. Syst. Assur. Eng. Manag., 1–10. 10.1007/s13198-022-01845-5

[B18] Presigny C. De Vico Fallani F. (2022). Colloquium: multiscale modeling of brain network organization. Rev. Mod. Phys. 94 (3), 031002. 10.1103/revmodphys.94.031002

[B19] Sakkalis V. (2011). Review of advanced techniques for the estimation of brain connectivity measured with EEG/MEG. Comput. Biol. Med. 41 (12), 1110–1117. 10.1016/j.compbiomed.2011.06.020 21794851

[B20] Sala A. Lizarraga A. Caminiti S. P. Calhoun V. D. Eickhoff S. B. Habeck C. (2022). Brain connectomics: time for a molecular imaging perspective? Trends Cognitive Sci. (99), 1–14. 10.1016/j.tics.2022.11.015 PMC10432882 36621368

[B21] Schirrmeister R. T. Springenberg J. T. Fiederer L. D. J. Glasstetter M. Eggensperger K. Tangermann M. (2017). Deep learning with convolutional neural networks for EEG decoding and visualization. Hum. brain Mapp. 38 (11), 5391–5420. 10.1002/hbm.23730 28782865 PMC5655781

[B22] Sharma R. R. Varshney P. Pachori R. B. Vishvakarma S. K. (2018). Automated system for epileptic EEG detection using iterative filtering. IEEE Sensors Lett. 2 (4), 1–4. 10.1109/lsens.2018.2882622

[B23] Sharma S. Shedsale A. Sharma R. R. (2023). Multivariate fast iterative filtering based automated system for grasp motor imagery identification using EEG signals. Int. J. Human–Computer Interact., 1–9. 10.1080/10447318.2023.2280327

[B24] Shi X. Chen Z. Wang H. Yeung D-Y. Wong W-K. Woo W-C. (2015). Convolutional LSTM network: a machine learning approach for precipitation nowcasting. Adv. neural Inf. Process. Syst., 28. 10.1007/978-3-319-21233-3_6

[B25] Shuting S. Xiaowei L. Jing Z. Wang Y. La R. Zhang X. (2019). Graph theory analysis of functional connectivity in major depression disorder with high-density resting state EEG data. IEEE Trans. neural Syst. rehabilitation Eng. 27 (3), 429–439. 10.1109/TNSRE.2019.2894423 30676968

[B26] Sola J. Sevilla J. (1997). Importance of input data normalization for the application of neural networks to complex industrial problems. IEEE Trans. Nucl. Sci. 44 (3), 1464–1468. 10.1109/23.589532

[B27] Sporns O. Tononi G. Kötter R. (2005). The human connectome: a structural description of the human brain. PLoS Comput. Biol. 1 (4), e42. 10.1371/journal.pcbi.0010042 16201007 PMC1239902

[B28] Thiebaut de Schotten M. Forkel S. J. (2022). The emergent properties of the connected brain. Science 378 (6619), 505–510. 10.1126/science.abq2591 36378968

[B29] van den Heuvel M. P. Hulshoff Pol H. E. (2010). Exploring the brain network: a review on resting-state f MRI functional connectivity. Eur. Neuropsychopharmacol. 20 (8), 519–534. 10.1016/j.euroneuro.2010.03.008 20471808

[B30] Wagh N. Varatharajah Y. (2020). Eeg-gcnn: augmenting electroencephalogram-based neurological disease diagnosis using a domain-guided graph convolutional neural network[C]//Machine Learning for Health. PMLR, 367–378.

[B31] Wang Z. Liu F. Shi S. Xia S. Peng F. Wang L. (2023). Automatic epileptic seizure detection based on persistent homology. Front. Physiology 14, 1227952. 10.3389/fphys.2023.1227952 PMC10773586 38192741

[B32] Yadati N. Nimishakavi M. Yadav P. (2019). Hypergcn: a new method for training graph convolutional networks on hypergraphs. Adv. Neural Inf. process Syst. 32. 10.48550/arXiv.1809.02589

[B33] Yan C.-G. Chen X. Li L. Castellanos F. X. Bai T. J. Bo Q. J. (2019). Reduced default mode network functional connectivity in patients with recurrent major depressive disorder. Proc. Natl. Acad. Sci. 116 (18), 9078–9083. 10.1073/pnas.1900390116 30979801 PMC6500168

[B34] Yang Y. Li F. Qin X. Wen H. Lin X. Huang D. (2023). Feature separation and adversarial training for the patient-independent detection of epileptic seizures. Front. Comput. Neurosci. 17, 1195334. 10.3389/fncom.2023.1195334 37538929 PMC10394297

[B35] Yang Y. Qin X. Lin X. Wen H. Peng Y. (2022). Epilepsy detection and analysis method for specific patient based on data augmentation and deep learning. Sheng Wu Yi Xue Gong Cheng Xue Za Zhi 39 (2), 293–300. 10.7507/1001-5515.202107060 35523550 PMC9927344

[B36] Yudhana A. Muslim A. Wati D. E. Puspitasari I. Azhari A. Mardhia M. M. (2020). Human emotion recognition based on EEG signal using fast fourier transform and K-Nearest neighbor. Adv. Sci. Technol. Eng. Syst. J. 5 (6), 1082–1088. 10.25046/aj0506131

[B37] Zabihi M. Kiranyaz S. Ince T. Gabbouj M. Patient-specific epileptic seizure detection in long-term EEG recording in paediatric patients with intractable seizures. 2013. Corpus ID: 62541685.

[B38] Zhang D. Yao L. Chen K. Wang S. Haghighi P. D. Sullivan C. (2019). A graph-based hierarchical attention model for movement intention detection from EEG signals. IEEE Trans. Neural Syst. Rehabilitation Eng. 27 (11), 2247–2253. 10.1109/TNSRE.2019.2943362 31562095

[B39] Zhang R. Sui L. Gong J. Cao J. (2023). EEG-based real-time diagnostic system with developed dynamic 2TEMD and dynamic ApEn algorithms. Front. Physiology 14, 1165450. 10.3389/fphys.2023.1165450 PMC10213912 37250115

[B40] Zhang X. Yao L. Dong M. Liu Z. Zhang Y. Li Y. (2020). Adversarial representation learning for robust patient-independent epileptic seizure detection. IEEE J. Biomed. health Inf. 24 (10), 2852–2859. 10.1109/JBHI.2020.2971610 32071011

[B41] Zhou M. Tian C. Cao R. Wang B. Niu Y. Hu T. (2018). Epileptic seizure detection based on EEG signals and CNN. Front. neuroinformatics 12, 95. 10.3389/fninf.2018.00095 PMC6295451 30618700

